# Bis[3,3-dimethyl-2-(2-oxoethyl­idene)indolinyl-κ^2^ *N*,*O*]palladium(II)

**DOI:** 10.1107/S1600536811022069

**Published:** 2011-06-18

**Authors:** Hamid Khaledi, Marilyn M. Olmstead, Hapipah Mohd Ali

**Affiliations:** a Department of Chemistry, University of Malaya, 50603 Kuala Lumpur, Malaysia; b Department of Chemistry, University of California, One Shields Avenue, Davis, CA, 95616, USA.

## Abstract

The asymmetric unit of the title compound, [Pd(C_12_H_12_NO)_2_], consists of three crystallographically independent half-mol­ecules. Each Pd^II^ atom lies on a center of inversion and is four-coordinated by two monoanionic forms of the amino­acryl­aldehyde in a square-planar geometry. In the crystal, adjacent mol­ecules are connected through C—H⋯π and C—H⋯O inter­actions into a three-dimensional polymeric structure.

## Related literature

For the structures of related compounds, see: Khaledi *et al.* (2011 ▶).
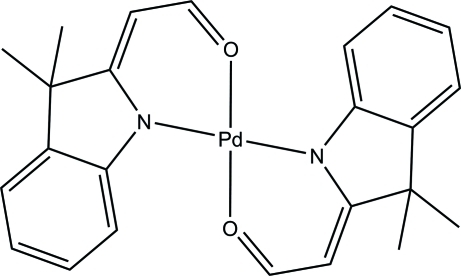


## Experimental

### 

#### Crystal data

[Pd(C_12_H_12_NO)_2_]
*M* *_r_* = 478.85
Triclinic, 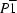

*a* = 10.453 (2) Å
*b* = 12.669 (3) Å
*c* = 13.056 (3) Å
α = 87.750 (3)°
β = 73.271 (3)°
γ = 70.565 (3)°
*V* = 1558.5 (6) Å^3^
*Z* = 3
Mo *K*α radiation
μ = 0.92 mm^−1^
*T* = 100 K
0.18 × 0.09 × 0.06 mm

#### Data collection

Bruker APEXII CCD diffractometer
Absorption correction: multi-scan (*SADABS*; Sheldrick, 1996 ▶) *T*_min_ = 0.853, *T*_max_ = 0.947
13381 measured reflections
6083 independent reflections
4905 reflections with *I* > 2σ(*I*)
*R*_int_ = 0.042

#### Refinement

*R*[*F* ^2^ > 2σ(*F* ^2^)] = 0.037
*wR*(*F* ^2^) = 0.095
*S* = 1.03
6083 reflections
403 parameters
H-atom parameters constrained
Δρ_max_ = 1.35 e Å^−3^
Δρ_min_ = −0.75 e Å^−3^

### 

Data collection: *APEX2* (Bruker, 2007 ▶); cell refinement: *SAINT* (Bruker, 2007 ▶); data reduction: *SAINT*; program(s) used to solve structure: *SHELXS97* (Sheldrick, 2008 ▶); program(s) used to refine structure: *SHELXL97* (Sheldrick, 2008 ▶); molecular graphics: *X-SEED* (Barbour, 2001 ▶); software used to prepare material for publication: *SHELXL97* and *publCIF* (Westrip, 2010 ▶).

## Supplementary Material

Crystal structure: contains datablock(s) I, global. DOI: 10.1107/S1600536811022069/is2718sup1.cif

Structure factors: contains datablock(s) I. DOI: 10.1107/S1600536811022069/is2718Isup2.hkl

Additional supplementary materials:  crystallographic information; 3D view; checkCIF report

## Figures and Tables

**Table 1:** Hydrogen-bond geometry (Å, °) *Cg*1 and *Cg*2 are the centroids of the benzene C27–C32 and C3–C8 rings, respectively.

*D*—H⋯*A*	*D*—H	H⋯*A*	*D*⋯*A*	*D*—H⋯*A*
C4—H4⋯O2^i^	0.95	2.40	3.353 (5)	177
C7—H7⋯O1^ii^	0.95	2.34	2.965 (4)	123
C19—H19⋯O2^i^	0.95	2.27	2.860 (5)	120
C31—H31⋯O3^iii^	0.95	2.25	2.861 (5)	121
C9—H9⋯*Cg*1^iv^	0.95	2.85	3.794 (5)	170
C33—H33⋯*Cg*2^i^	0.95	2.76	3.630 (4)	153

Symmetry codes: (i) 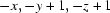; (ii) 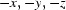; (iii) 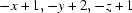; (iv) 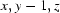.

**Fig. 1.**
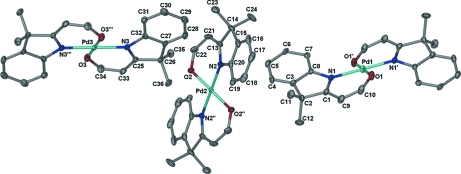
The molecular structure of the title compound (50% probability ellipsoids). Hydrogen atoms have been omitted for clarity. Symmetry codes: ' = -x, -y, -z; '' = -x, -y+1, -z+1; ''' = -x+1, -y+2, -z+1.

**Fig. 2.**
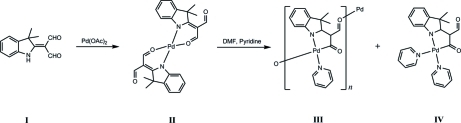
Reaction scheme.

## supplementary crystallographic information

### Comment

We have recently reported the reaction of 2-(diformylmethylidene)-3,3-dimethylindole, I, (Fig. 2), with palladium(II) ion to produce the complex II (Khaledi *et al.*, 2011). As was reported, further recrystallization of the obtained *N*_2_*O*_2_-coordinated Pd^II^ complex from a mixture of DMF and pyridine led to the formation of acyl-palladium complexes III & IV. We were surprised to discover that leaving the recrystallization solution at room temperature for a longer time gave rise to slow disappearance of crystals of III & IV and formation of the orange crystals of the title compound. Herein, we report the crystal structure of the obtained Pd^II^ complex.

The crystal structure is composed of three square planar centrosymmetric complexes. In each molecule, two monoanionic ligands chelate the Pd^II^ ion in an *N,O*-mode to form two six-membered rings with the metal atom. The O—Pd—N bite angles are 90.38 (11), 90.13 (11) and 91.08 (11)° for the complexes of Pd1, Pd2 and Pd3, respectively. The three complexes have little difference in their geometries, but differ more in their intermolecular interactions. The Pd1 and Pd3 complexes are connected through C—H···π interactions (Table 1), forming infinite layers parallel to the *ac* plane. The layers are further linked into a three dimensional network *via* C—H···O hydrogen bonds (Table 1) formed between Pd1 and Pd2 molecules. Intramolecular C—H···O hydrogen bonding occurs in all three complexes.

### Experimental

A solution of palladium(II) complex II (Fig. 2) in a mixture of DMF and pyridine yielded the yellowish green crystals of III and IV in two weeks. Upon standing of the solution in a closed vial at room temperature for a month, the orange crystals of the title compound appeared while the former crystals began to disappear slowly and finally disappeared completely after two months.

### Refinement

Hydrogen atoms were placed at calculated positions and refined as riding atoms with distances of H—C(*sp*^2^) = 0.95 and H—C(methyl) = 0.98 Å and with *U*_iso_(H) set to 1.2 (1.5 for methyl)*U*_eq_(C). The most disagreeable reflections with delta(*F^2^*)/e.s.d. >10 were omitted (13 reflections). The maximum and minimum residual electron density peaks of 1.35 Å and -0.75 e Å^-3^, respectively, were located 1.44 Å and 0.85 Å from the H16 and Pd2 atoms, respectively.

### Crystal data

**Table:**

[Pd(C_12_H_12_NO)_2_]	*Z* = 3
*M**_r_* = 478.85	*F*(000) = 732
Triclinic, *P*1	*D*_x_ = 1.531 Mg m^−^^3^
Hall symbol: -P 1	Mo *K*α radiation, λ = 0.71073 Å
*a* = 10.453 (2) Å	Cell parameters from 2716 reflections
*b* = 12.669 (3) Å	θ = 2.3–24.8°
*c* = 13.056 (3) Å	µ = 0.92 mm^−^^1^
α = 87.750 (3)°	*T* = 100 K
β = 73.271 (3)°	Rod, orange
γ = 70.565 (3)°	0.18 × 0.09 × 0.06 mm
*V* = 1558.5 (6) Å^3^	

### Data collection

**Table:**

Bruker APEXII CCD diffractometer	6083 independent reflections
Radiation source: fine-focus sealed tube	4905 reflections with *I* > 2σ(*I*)
graphite	*R*_int_ = 0.042
φ and ω scans	θ_max_ = 26.0°, θ_min_ = 2.2°
Absorption correction: multi-scan (*SADABS*; Sheldrick, 1996)	*h* = −12→12
*T*_min_ = 0.853, *T*_max_ = 0.947	*k* = −15→15
13381 measured reflections	*l* = −16→16

### Refinement

**Table:**

Refinement on *F*^2^	Primary atom site location: structure-invariant direct methods
Least-squares matrix: full	Secondary atom site location: difference Fourier map
*R*[*F*^2^ > 2σ(*F*^2^)] = 0.037	Hydrogen site location: inferred from neighbouring sites
*wR*(*F*^2^) = 0.095	H-atom parameters constrained
*S* = 1.03	*w* = 1/[σ^2^(*F*_o_^2^) + (0.042*P*)^2^] where *P* = (*F*_o_^2^ + 2*F*_c_^2^)/3
6083 reflections	(Δ/σ)_max_ < 0.001
403 parameters	Δρ_max_ = 1.35 e Å^−^^3^
0 restraints	Δρ_min_ = −0.75 e Å^−^^3^

### Special details

**Table:**

Geometry. All e.s.d.'s (except the e.s.d. in the dihedral angle between two l.s. planes) are estimated using the full covariance matrix. The cell e.s.d.'s are taken into account individually in the estimation of e.s.d.'s in distances, angles and torsion angles; correlations between e.s.d.'s in cell parameters are only used when they are defined by crystal symmetry. An approximate (isotropic) treatment of cell e.s.d.'s is used for estimating e.s.d.'s involving l.s. planes.
Refinement. Refinement of *F*^2^ against ALL reflections. The weighted *R*-factor *wR* and goodness of fit *S* are based on *F*^2^, conventional *R*-factors *R* are based on *F*, with *F* set to zero for negative *F*^2^. The threshold expression of *F*^2^ > σ(*F*^2^) is used only for calculating *R*-factors(gt) *etc*. and is not relevant to the choice of reflections for refinement. *R*-factors based on *F*^2^ are statistically about twice as large as those based on *F*, and *R*- factors based on ALL data will be even larger.

### Fractional atomic coordinates and isotropic or equivalent isotropic displacement parameters (Å^2^)

**Table:**

	*x*	*y*	*z*	*U*_iso_*/*U*_eq_	
Pd1	0.0000	0.0000	0.0000	0.01805 (11)	
O1	0.2061 (3)	−0.0799 (2)	−0.0656 (2)	0.0239 (6)	
N1	0.0435 (3)	0.0856 (2)	0.1068 (2)	0.0179 (7)	
C1	0.1611 (4)	0.0445 (3)	0.1357 (3)	0.0202 (8)	
C2	0.1613 (4)	0.1157 (3)	0.2271 (3)	0.0190 (8)	
C3	0.0286 (4)	0.2125 (3)	0.2365 (3)	0.0190 (8)	
C4	−0.0276 (4)	0.3108 (3)	0.2998 (3)	0.0212 (9)	
H4	0.0172	0.3235	0.3497	0.025*	
C5	−0.1499 (4)	0.3907 (3)	0.2897 (3)	0.0229 (9)	
H5	−0.1900	0.4589	0.3329	0.027*	
C6	−0.2146 (4)	0.3718 (3)	0.2166 (3)	0.0214 (9)	
H6	−0.2977	0.4280	0.2092	0.026*	
C7	−0.1596 (4)	0.2716 (3)	0.1537 (3)	0.0185 (8)	
H7	−0.2050	0.2585	0.1045	0.022*	
C8	−0.0379 (4)	0.1920 (3)	0.1645 (3)	0.0156 (8)	
C9	0.2790 (4)	−0.0489 (3)	0.0843 (3)	0.0261 (9)	
H9	0.3503	−0.0801	0.1191	0.031*	
C10	0.2965 (4)	−0.0971 (3)	−0.0124 (3)	0.0272 (10)	
H10	0.3874	−0.1504	−0.0448	0.033*	
C11	0.2927 (4)	0.1515 (3)	0.2003 (4)	0.0285 (10)	
H11A	0.2987	0.1924	0.1348	0.043*	
H11B	0.3778	0.0848	0.1893	0.043*	
H11C	0.2859	0.2001	0.2596	0.043*	
C12	0.1514 (5)	0.0497 (4)	0.3293 (3)	0.0293 (10)	
H12A	0.1449	0.0972	0.3893	0.044*	
H12B	0.2362	−0.0173	0.3176	0.044*	
H12C	0.0669	0.0272	0.3457	0.044*	
Pd2	0.0000	0.5000	0.5000	0.01294 (11)	
O2	−0.1393 (3)	0.6550 (2)	0.52634 (19)	0.0188 (6)	
N2	0.0867 (3)	0.5334 (3)	0.3486 (2)	0.0159 (7)	
C13	0.0131 (4)	0.6108 (3)	0.2965 (3)	0.0193 (8)	
C14	0.0925 (4)	0.6054 (3)	0.1789 (3)	0.0235 (9)	
C15	0.2302 (4)	0.5152 (3)	0.1742 (3)	0.0230 (9)	
C16	0.3528 (5)	0.4708 (4)	0.0914 (3)	0.0310 (11)	
H16	0.3586	0.4957	0.0211	0.037*	
C17	0.4673 (5)	0.3894 (4)	0.1122 (4)	0.0350 (11)	
H17	0.5513	0.3564	0.0552	0.042*	
C18	0.4609 (4)	0.3559 (3)	0.2136 (4)	0.0301 (10)	
H18	0.5417	0.3014	0.2264	0.036*	
C19	0.3392 (4)	0.3996 (3)	0.2981 (3)	0.0214 (9)	
H19	0.3357	0.3768	0.3688	0.026*	
C20	0.2232 (4)	0.4774 (3)	0.2763 (3)	0.0188 (8)	
C21	−0.1195 (4)	0.6926 (3)	0.3431 (3)	0.0253 (9)	
H21	−0.1702	0.7359	0.2973	0.030*	
C22	−0.1785 (4)	0.7127 (3)	0.4509 (3)	0.0248 (9)	
H22	−0.2596	0.7783	0.4734	0.030*	
C23	0.1133 (5)	0.7192 (4)	0.1471 (3)	0.0328 (11)	
H23A	0.1568	0.7420	0.1957	0.049*	
H23B	0.0210	0.7762	0.1521	0.049*	
H23C	0.1752	0.7112	0.0734	0.049*	
C24	0.0148 (5)	0.5727 (4)	0.1081 (3)	0.0373 (12)	
H24A	0.0701	0.5674	0.0326	0.056*	
H24B	−0.0789	0.6300	0.1189	0.056*	
H24C	0.0037	0.5002	0.1277	0.056*	
Pd3	0.5000	1.0000	0.5000	0.01505 (11)	
O3	0.3211 (3)	1.0191 (2)	0.6163 (2)	0.0218 (6)	
N3	0.4648 (3)	0.8844 (2)	0.4194 (2)	0.0171 (7)	
C25	0.3843 (4)	0.8253 (3)	0.4695 (3)	0.0170 (8)	
C26	0.3941 (4)	0.7294 (3)	0.3976 (3)	0.0210 (9)	
C27	0.4854 (4)	0.7514 (3)	0.2935 (3)	0.0205 (9)	
C28	0.5324 (5)	0.6965 (3)	0.1924 (3)	0.0267 (10)	
H28	0.5075	0.6331	0.1816	0.032*	
C29	0.6159 (5)	0.7355 (4)	0.1080 (3)	0.0310 (10)	
H29	0.6506	0.6978	0.0390	0.037*	
C30	0.6491 (4)	0.8297 (4)	0.1241 (3)	0.0277 (10)	
H30	0.7044	0.8568	0.0650	0.033*	
C31	0.6035 (4)	0.8854 (3)	0.2245 (3)	0.0218 (9)	
H31	0.6264	0.9500	0.2346	0.026*	
C32	0.5235 (4)	0.8437 (3)	0.3091 (3)	0.0181 (8)	
C33	0.2959 (4)	0.8471 (3)	0.5760 (3)	0.0228 (9)	
H33	0.2549	0.7924	0.6067	0.027*	
C34	0.2652 (4)	0.9417 (3)	0.6379 (3)	0.0253 (9)	
H34	0.1943	0.9513	0.7048	0.030*	
C35	0.2477 (4)	0.7321 (4)	0.3930 (3)	0.0271 (10)	
H35A	0.1988	0.8047	0.3691	0.041*	
H35B	0.1914	0.7216	0.4644	0.041*	
H35C	0.2595	0.6719	0.3426	0.041*	
C36	0.4685 (5)	0.6181 (3)	0.4390 (3)	0.0288 (10)	
H36A	0.4821	0.5561	0.3900	0.043*	
H36B	0.4102	0.6096	0.5104	0.043*	
H36C	0.5609	0.6173	0.4432	0.043*	

### Atomic displacement parameters (Å^2^)

**Table:**

	*U*^11^	*U*^22^	*U*^33^	*U*^12^	*U*^13^	*U*^23^
Pd1	0.0179 (2)	0.0176 (2)	0.0192 (2)	−0.00839 (18)	−0.00271 (18)	−0.00333 (17)
O1	0.0185 (14)	0.0241 (15)	0.0265 (15)	−0.0085 (12)	0.0006 (12)	−0.0092 (12)
N1	0.0220 (17)	0.0140 (16)	0.0178 (16)	−0.0082 (14)	−0.0034 (14)	0.0003 (13)
C1	0.022 (2)	0.016 (2)	0.027 (2)	−0.0102 (17)	−0.0078 (18)	0.0025 (17)
C2	0.021 (2)	0.020 (2)	0.021 (2)	−0.0119 (17)	−0.0078 (17)	0.0004 (16)
C3	0.019 (2)	0.0149 (19)	0.024 (2)	−0.0069 (16)	−0.0056 (17)	0.0017 (16)
C4	0.024 (2)	0.022 (2)	0.021 (2)	−0.0137 (18)	−0.0065 (17)	−0.0036 (17)
C5	0.030 (2)	0.018 (2)	0.022 (2)	−0.0134 (18)	−0.0019 (18)	−0.0021 (17)
C6	0.023 (2)	0.0128 (19)	0.027 (2)	−0.0051 (17)	−0.0063 (18)	0.0028 (17)
C7	0.023 (2)	0.020 (2)	0.0153 (19)	−0.0124 (17)	−0.0038 (16)	0.0035 (16)
C8	0.020 (2)	0.0148 (19)	0.0134 (18)	−0.0104 (16)	−0.0004 (16)	0.0002 (15)
C9	0.023 (2)	0.021 (2)	0.039 (3)	−0.0107 (18)	−0.013 (2)	0.0015 (19)
C10	0.017 (2)	0.020 (2)	0.044 (3)	−0.0069 (18)	−0.005 (2)	−0.0080 (19)
C11	0.021 (2)	0.028 (2)	0.042 (3)	−0.0128 (19)	−0.012 (2)	0.000 (2)
C12	0.033 (2)	0.029 (2)	0.030 (2)	−0.010 (2)	−0.018 (2)	0.0124 (19)
Pd2	0.0149 (2)	0.0131 (2)	0.0107 (2)	−0.00434 (17)	−0.00404 (16)	0.00026 (15)
O2	0.0198 (14)	0.0162 (14)	0.0180 (14)	−0.0028 (11)	−0.0060 (11)	0.0018 (11)
N2	0.0183 (17)	0.0186 (17)	0.0125 (15)	−0.0080 (14)	−0.0049 (13)	0.0009 (13)
C13	0.024 (2)	0.026 (2)	0.0143 (19)	−0.0140 (18)	−0.0089 (17)	0.0046 (16)
C14	0.033 (2)	0.036 (2)	0.0104 (18)	−0.023 (2)	−0.0078 (17)	0.0071 (17)
C15	0.030 (2)	0.026 (2)	0.019 (2)	−0.0220 (19)	−0.0021 (18)	−0.0027 (17)
C16	0.045 (3)	0.035 (3)	0.016 (2)	−0.029 (2)	0.007 (2)	−0.0068 (19)
C17	0.031 (3)	0.024 (2)	0.042 (3)	−0.016 (2)	0.013 (2)	−0.010 (2)
C18	0.023 (2)	0.023 (2)	0.043 (3)	−0.0139 (19)	0.000 (2)	−0.002 (2)
C19	0.021 (2)	0.018 (2)	0.027 (2)	−0.0114 (17)	−0.0031 (18)	0.0005 (17)
C20	0.022 (2)	0.021 (2)	0.0172 (19)	−0.0156 (18)	−0.0006 (16)	−0.0042 (16)
C21	0.027 (2)	0.025 (2)	0.025 (2)	−0.0058 (19)	−0.0139 (19)	0.0110 (18)
C22	0.020 (2)	0.017 (2)	0.031 (2)	0.0026 (17)	−0.0089 (19)	0.0024 (18)
C23	0.049 (3)	0.036 (3)	0.023 (2)	−0.026 (2)	−0.013 (2)	0.014 (2)
C24	0.052 (3)	0.059 (3)	0.021 (2)	−0.037 (3)	−0.021 (2)	0.013 (2)
Pd3	0.0166 (2)	0.0113 (2)	0.0193 (2)	−0.00579 (17)	−0.00698 (17)	0.00068 (16)
O3	0.0213 (15)	0.0200 (15)	0.0245 (15)	−0.0110 (12)	−0.0020 (12)	−0.0023 (12)
N3	0.0196 (17)	0.0142 (16)	0.0197 (16)	−0.0063 (14)	−0.0083 (14)	0.0016 (13)
C25	0.020 (2)	0.0133 (19)	0.021 (2)	−0.0072 (16)	−0.0090 (17)	0.0022 (16)
C26	0.029 (2)	0.014 (2)	0.024 (2)	−0.0088 (17)	−0.0106 (18)	0.0022 (16)
C27	0.027 (2)	0.0103 (19)	0.025 (2)	−0.0031 (17)	−0.0137 (18)	0.0014 (16)
C28	0.037 (3)	0.017 (2)	0.030 (2)	−0.0087 (19)	−0.015 (2)	−0.0028 (18)
C29	0.043 (3)	0.030 (2)	0.024 (2)	−0.012 (2)	−0.015 (2)	0.0016 (19)
C30	0.028 (2)	0.032 (2)	0.021 (2)	−0.007 (2)	−0.0073 (19)	0.0017 (19)
C31	0.025 (2)	0.017 (2)	0.026 (2)	−0.0091 (18)	−0.0093 (18)	0.0017 (17)
C32	0.020 (2)	0.017 (2)	0.020 (2)	−0.0031 (16)	−0.0123 (17)	0.0001 (16)
C33	0.028 (2)	0.018 (2)	0.027 (2)	−0.0143 (18)	−0.0076 (18)	0.0064 (17)
C34	0.023 (2)	0.029 (2)	0.022 (2)	−0.0137 (19)	0.0017 (18)	0.0004 (18)
C35	0.030 (2)	0.029 (2)	0.033 (2)	−0.019 (2)	−0.015 (2)	0.0028 (19)
C36	0.040 (3)	0.013 (2)	0.035 (2)	−0.0062 (19)	−0.017 (2)	0.0040 (18)

### Geometric parameters (Å, °)

**Table:**

Pd1—O1^i^	1.989 (3)	C17—C18	1.364 (6)
Pd1—O1	1.989 (3)	C17—H17	0.9500
Pd1—N1^i^	2.035 (3)	C18—C19	1.384 (5)
Pd1—N1	2.035 (3)	C18—H18	0.9500
O1—C10	1.285 (5)	C19—C20	1.381 (5)
N1—C1	1.323 (5)	C19—H19	0.9500
N1—C8	1.434 (4)	C21—C22	1.359 (5)
C1—C9	1.410 (5)	C21—H21	0.9500
C1—C2	1.524 (5)	C22—H22	0.9500
C2—C3	1.491 (5)	C23—H23A	0.9800
C2—C11	1.530 (5)	C23—H23B	0.9800
C2—C12	1.543 (5)	C23—H23C	0.9800
C3—C4	1.377 (5)	C24—H24A	0.9800
C3—C8	1.397 (5)	C24—H24B	0.9800
C4—C5	1.379 (5)	C24—H24C	0.9800
C4—H4	0.9500	Pd3—O3^iii^	1.988 (3)
C5—C6	1.387 (5)	Pd3—O3	1.988 (3)
C5—H5	0.9500	Pd3—N3	2.022 (3)
C6—C7	1.393 (5)	Pd3—N3^iii^	2.022 (3)
C6—H6	0.9500	O3—C34	1.284 (4)
C7—C8	1.375 (5)	N3—C25	1.327 (5)
C7—H7	0.9500	N3—C32	1.433 (5)
C9—C10	1.361 (5)	C25—C33	1.408 (5)
C9—H9	0.9500	C25—C26	1.524 (5)
C10—H10	0.9500	C26—C27	1.495 (5)
C11—H11A	0.9800	C26—C36	1.529 (5)
C11—H11B	0.9800	C26—C35	1.537 (5)
C11—H11C	0.9800	C27—C28	1.392 (5)
C12—H12A	0.9800	C27—C32	1.395 (5)
C12—H12B	0.9800	C28—C29	1.382 (6)
C12—H12C	0.9800	C28—H28	0.9500
Pd2—O2^ii^	1.989 (2)	C29—C30	1.387 (6)
Pd2—O2	1.989 (2)	C29—H29	0.9500
Pd2—N2^ii^	2.016 (3)	C30—C31	1.390 (5)
Pd2—N2	2.016 (3)	C30—H30	0.9500
O2—C22	1.285 (4)	C31—C32	1.383 (5)
N2—C13	1.328 (5)	C31—H31	0.9500
N2—C20	1.426 (5)	C33—C34	1.365 (5)
C13—C21	1.405 (5)	C33—H33	0.9500
C13—C14	1.512 (5)	C34—H34	0.9500
C14—C15	1.497 (6)	C35—H35A	0.9800
C14—C24	1.540 (5)	C35—H35B	0.9800
C14—C23	1.552 (5)	C35—H35C	0.9800
C15—C16	1.378 (5)	C36—H36A	0.9800
C15—C20	1.388 (5)	C36—H36B	0.9800
C16—C17	1.384 (6)	C36—H36C	0.9800
C16—H16	0.9500		
			
O1^i^—Pd1—O1	180.00 (10)	C17—C18—C19	121.4 (4)
O1^i^—Pd1—N1^i^	90.38 (11)	C17—C18—H18	119.3
O1—Pd1—N1^i^	89.62 (11)	C19—C18—H18	119.3
O1^i^—Pd1—N1	89.62 (11)	C20—C19—C18	117.7 (4)
O1—Pd1—N1	90.38 (11)	C20—C19—H19	121.1
N1^i^—Pd1—N1	180.0	C18—C19—H19	121.1
C10—O1—Pd1	122.2 (3)	C19—C20—C15	121.3 (4)
C1—N1—C8	108.0 (3)	C19—C20—N2	128.4 (3)
C1—N1—Pd1	121.8 (2)	C15—C20—N2	110.3 (3)
C8—N1—Pd1	130.2 (2)	C22—C21—C13	123.0 (4)
N1—C1—C9	125.1 (4)	C22—C21—H21	118.5
N1—C1—C2	112.2 (3)	C13—C21—H21	118.5
C9—C1—C2	122.5 (3)	O2—C22—C21	128.7 (4)
C3—C2—C1	100.7 (3)	O2—C22—H22	115.6
C3—C2—C11	112.4 (3)	C21—C22—H22	115.6
C1—C2—C11	112.4 (3)	C14—C23—H23A	109.5
C3—C2—C12	111.7 (3)	C14—C23—H23B	109.5
C1—C2—C12	109.3 (3)	H23A—C23—H23B	109.5
C11—C2—C12	110.1 (3)	C14—C23—H23C	109.5
C4—C3—C8	121.0 (4)	H23A—C23—H23C	109.5
C4—C3—C2	130.2 (4)	H23B—C23—H23C	109.5
C8—C3—C2	108.8 (3)	C14—C24—H24A	109.5
C3—C4—C5	118.9 (4)	C14—C24—H24B	109.5
C3—C4—H4	120.5	H24A—C24—H24B	109.5
C5—C4—H4	120.5	C14—C24—H24C	109.5
C4—C5—C6	120.2 (4)	H24A—C24—H24C	109.5
C4—C5—H5	119.9	H24B—C24—H24C	109.5
C6—C5—H5	119.9	O3^iii^—Pd3—O3	180.000 (1)
C5—C6—C7	121.0 (4)	O3^iii^—Pd3—N3	88.92 (11)
C5—C6—H6	119.5	O3—Pd3—N3	91.08 (11)
C7—C6—H6	119.5	O3^iii^—Pd3—N3^iii^	91.08 (11)
C8—C7—C6	118.5 (3)	O3—Pd3—N3^iii^	88.92 (11)
C8—C7—H7	120.7	N3—Pd3—N3^iii^	179.999 (1)
C6—C7—H7	120.7	C34—O3—Pd3	122.8 (2)
C7—C8—C3	120.2 (3)	C25—N3—C32	108.0 (3)
C7—C8—N1	129.8 (3)	C25—N3—Pd3	121.4 (2)
C3—C8—N1	109.9 (3)	C32—N3—Pd3	130.4 (2)
C10—C9—C1	123.7 (4)	N3—C25—C33	125.6 (3)
C10—C9—H9	118.1	N3—C25—C26	112.3 (3)
C1—C9—H9	118.1	C33—C25—C26	122.1 (3)
O1—C10—C9	128.5 (4)	C27—C26—C25	100.5 (3)
O1—C10—H10	115.7	C27—C26—C36	111.4 (3)
C9—C10—H10	115.7	C25—C26—C36	108.9 (3)
C2—C11—H11A	109.5	C27—C26—C35	113.6 (3)
C2—C11—H11B	109.5	C25—C26—C35	112.3 (3)
H11A—C11—H11B	109.5	C36—C26—C35	109.7 (3)
C2—C11—H11C	109.5	C28—C27—C32	120.2 (4)
H11A—C11—H11C	109.5	C28—C27—C26	130.8 (4)
H11B—C11—H11C	109.5	C32—C27—C26	109.0 (3)
C2—C12—H12A	109.5	C29—C28—C27	119.1 (4)
C2—C12—H12B	109.5	C29—C28—H28	120.4
H12A—C12—H12B	109.5	C27—C28—H28	120.4
C2—C12—H12C	109.5	C28—C29—C30	120.0 (4)
H12A—C12—H12C	109.5	C28—C29—H29	120.0
H12B—C12—H12C	109.5	C30—C29—H29	120.0
O2^ii^—Pd2—O2	180.0	C29—C30—C31	121.7 (4)
O2^ii^—Pd2—N2^ii^	90.13 (11)	C29—C30—H30	119.1
O2—Pd2—N2^ii^	89.87 (11)	C31—C30—H30	119.1
O2^ii^—Pd2—N2	89.87 (11)	C32—C31—C30	117.8 (4)
O2—Pd2—N2	90.13 (11)	C32—C31—H31	121.1
N2^ii^—Pd2—N2	179.999 (1)	C30—C31—H31	121.1
C22—O2—Pd2	122.6 (2)	C31—C32—C27	121.1 (3)
C13—N2—C20	108.2 (3)	C31—C32—N3	129.0 (3)
C13—N2—Pd2	122.1 (2)	C27—C32—N3	109.9 (3)
C20—N2—Pd2	129.4 (2)	C34—C33—C25	124.3 (4)
N2—C13—C21	125.5 (3)	C34—C33—H33	117.8
N2—C13—C14	111.9 (3)	C25—C33—H33	117.8
C21—C13—C14	122.6 (3)	O3—C34—C33	127.6 (4)
C15—C14—C13	101.2 (3)	O3—C34—H34	116.2
C15—C14—C24	111.8 (3)	C33—C34—H34	116.2
C13—C14—C24	111.2 (3)	C26—C35—H35A	109.5
C15—C14—C23	111.8 (3)	C26—C35—H35B	109.5
C13—C14—C23	110.8 (3)	H35A—C35—H35B	109.5
C24—C14—C23	109.9 (3)	C26—C35—H35C	109.5
C16—C15—C20	119.8 (4)	H35A—C35—H35C	109.5
C16—C15—C14	131.8 (4)	H35B—C35—H35C	109.5
C20—C15—C14	108.3 (3)	C26—C36—H36A	109.5
C15—C16—C17	118.9 (4)	C26—C36—H36B	109.5
C15—C16—H16	120.5	H36A—C36—H36B	109.5
C17—C16—H16	120.5	C26—C36—H36C	109.5
C18—C17—C16	120.7 (4)	H36A—C36—H36C	109.5
C18—C17—H17	119.6	H36B—C36—H36C	109.5
C16—C17—H17	119.6		

Symmetry codes: (i) −*x*, −*y*, −*z*; (ii) −*x*, −*y*+1, −*z*+1; (iii) −*x*+1, −*y*+2, −*z*+1.

### Hydrogen-bond geometry (Å, °)

**Table:**

*Cg*1 and *Cg*2 are the centroids of the benzene C27–C32 and C3–C8 rings, respectively.

**Table:**

*D*—H···*A*	*D*—H	H···*A*	*D*···*A*	*D*—H···*A*
C4—H4···O2^ii^	0.95	2.40	3.353 (5)	177
C7—H7···O1^i^	0.95	2.34	2.965 (4)	123
C19—H19···O2^ii^	0.95	2.27	2.860 (5)	120
C31—H31···O3^iii^	0.95	2.25	2.861 (5)	121
C9—H9···Cg1^iv^	0.95	2.85	3.794 (5)	170
C33—H33···Cg2^ii^	0.95	2.76	3.630 (4)	153

Symmetry codes: (ii) −*x*, −*y*+1, −*z*+1; (i) −*x*, −*y*, −*z*; (iii) −*x*+1, −*y*+2, −*z*+1; (iv) *x*, *y*−1, *z*.
